# Reading virtual slide using web viewers: results of subjective experience with three different solutions

**DOI:** 10.1186/1746-1596-3-S1-S23

**Published:** 2008-07-15

**Authors:** Marcial García Rojo, Antonio J Gallardo, Lucía González, Carlos Peces, Cristina Murillo, Jesús González, Jose Sacristán

**Affiliations:** 1 Hospital General de Ciudad Real. Calle Tomelloso s/n. 13004 Ciudad Real, Spain; 2 Information Technologies Department of the Regional Health Care Services of Castilla-La Mancha (SESCAM). Calle Huerfanos Cristinos 5, 47071 Toledo, Spain

## Abstract

**Background:**

Virtual slides are viewed using interactive software that enables the user to simulate the behaviour of a conventional optical microscope, like adjusting magnifications and navigating to any portion of the image. Nowadays, information about the performance and features of web-based solutions for reading slides in real environments is still scarce. The objective of this study is analyzing the subjective experience of pathologists with virtual slides, comparing the time needed to read slides using different web viewers and different network connections.

**Methods:**

Eight slides were randomly selected (4 biopsies and 2 cytologies) from Hospital General de Ciudad Real (HGCR) archives. Three different virtual slide web-viewing solutions were analyzed: Aperio web server, Olympus NetImage Server, and Aurora mScope. Five pathologists studied to time needed to access images of each virtual slide, selecting a panoramic view, 10 low magnification fields, and 20 high magnification fields.

**Results:**

Aperio viewer is very efficient in overview images. Aurora viewer is especially efficient in lower magnifications (10×). For larger magnifications (20× and 40×) no significant differences were found between different vendors. Olympus was found to be the most user-friendly interface. When comparing Internet with intranet connections, despite being slower, users also felt comfortable using virtual slides through Internet connection.

**Conclusion:**

Available web solutions for virtual slides have different advantages, mainly in functionalities and optimization for different magnifications. Pathologist should select the solutions adapted to their needs.

## Background

Virtual slides, also known as digital slides or whole slide images (WSIs) are usually viewed using interactive software "virtual microscopy" which enables the user to simulate the behaviour of a conventional optical microscope, like adjusting magnifications and navigating to any portion of the image [1]. Nowadays, information about the performance and features of in real environments is still scarce.

## Methods

This is a transversal study with biopsies and cytologies obtained from the Hospital General de Ciudad Real (HGCR) archives. Eight slides (see Table 1) were randomly selected (6 biopsies and 2 cytologies). They were digitized using Aperio Scanscope XT (40×, JPEG200 compression, version 8) and Olympus SIS dotslide (40×, CMW compression, version 1.2 build 2992). Virtual slide files (Aperio SVS files and Olympus VSI files) were copied to two different servers (external servers and hospital server), each of them running 3 different web servers for virtual slides (Aperio web server, Olympus SIS NetImage Server, and Aurora mScope). Aurora mScope was used to access both Aperio SVS files and Olympus VSI files.

**Table 1:** File information of virtual slides scanned at 40×

**Number**	**Specimen**	**Stain**	**Dimensions in pixels**	**Compressed file size**
1	Soft tissue biopsy	Vimentin	39,360 × 38,954	379,46 MB
2	Bronchial smear	Papanicolau	209,280 × 82,122	364,62 MB
3	Monolayer cervical cytology	Papanicolau	89,280 × 86,555	237,96 MB
4	Lymph node biopsy	H&E	141,739 × 69,655	721,1 MB
5	Skin biopsy	H&E	149,563 × 112,227	811,32 MB
6	Nose biopsy	H&E	68,160 × 42,439	394,42 MB
7	Lung biosy	H&E	81,600 × 48,491	628.52 MB
8	Testis biossy	H&E	73,920 × 48,002	425,13 MB

Five pathologists studied to time needed to access images of each virtual slide, selecting a panoramic view, 10 low magnification fields, and 20 high magnification fields. In all cases, images were shown with a maximized window. All times were recorded in seconds.

An external server, a multi-domain dedicated server of the Spanish Society of Pathology running Aurora mScope server  was used. The Olympus SIS NetImage Server was installed in a server located at: 

Internet Aperio web server is available at 

Each pathologist accessed all eight images both using the HGCR intranet 1 GB Ethernet connection and residential Internet connection. Residential Asymmetric Digital Subscriber Line (ADSL) was 2520 Kbps/270 Kbps (mean values).

In the hospital environment, the same computer was used (Fujitsu Siemens Esprimo E, AMD Sempron Processor 3600+, 1 GB RAM, Serial ATA II hard disk, 17" scenic view A17-2 monitor). In the residential environment, each pathologist used a different computer (from notebooks with 512 GB RAM with a 15" screen to high performance desktop computers (CPU Intel Core2 6700 2.66 GHz, 3 GB RAM, Serial ATA-300 7200 RPM hard disk with a 21" monitor). All web viewers were run using Microsoft Internet Explorer in Windows XP operating system.

All measurements were made using a screen size of 1024 × 768 pixels.

The following variables were recorded twice (first with intranet servers and then accessing external servers using residential ADSL connection), using maximized/full screen window:

1. Time to show complete virtual slide panoramic view (default overview)

2. Time to show complete 10× image (recording 10 fields)

3. Time to show complete 20× image (recording 10 fields)

4. Time to show complete 40× image (recording 10 fields)

Quantitative data analysis was performed using Kirkman's tools [2] and confirmed with the SPSS statistical package (version for SESCAM). Means and SDs of the differences were then calculated, and the corresponding paired t tests were performed. Statistical significance was assumed at a P value of less than or equal to 0.05.

## Results

### Virtual slides viewers

**Figure 1:**
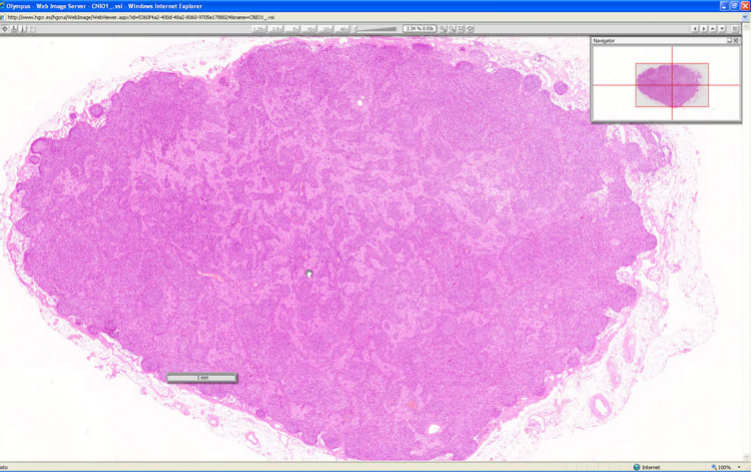
**Olympus virtual slide web viewer**. Olympus viewer is a user friendly viewer based in a Flash plug-in.

**Figure 2:**
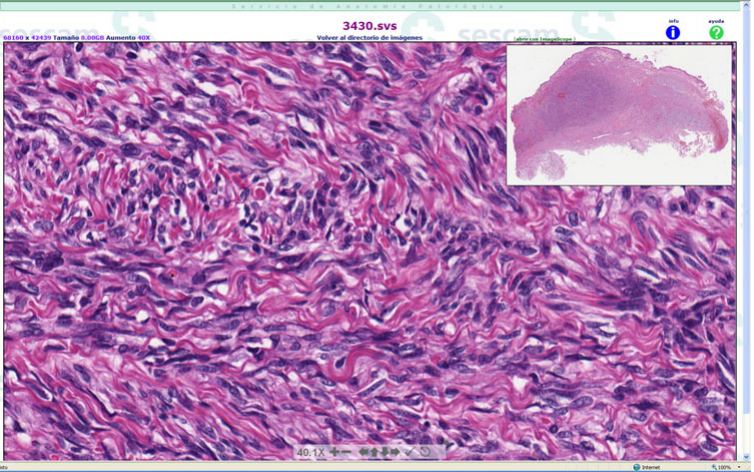
**Aperio virtual slide viewer**. Aperio web viewer is based in Zoomify viewer that reads Aperio SVS files.

**Figure 3:**
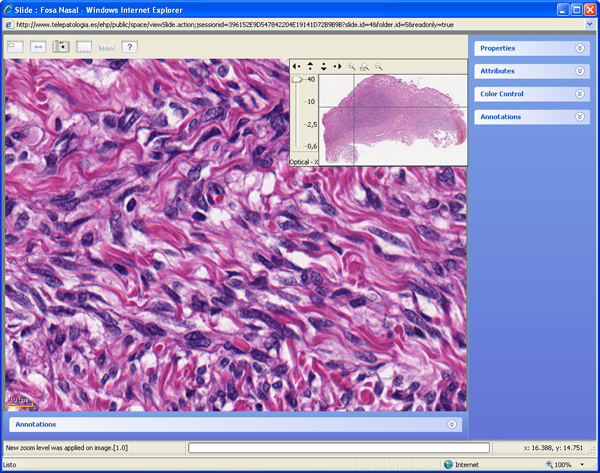
**Aurora virtual slide viewer**. Aurora java applet allows reading multiple file formats (SVS, VSI, DICOM, etc.).

User interface of the three virtual web viewers are quite different. Olympus (Figure 1) and Aperio (Figure 2) web viewers are based on Adobe Flash^© ^plug-in, and Aurora viewer (Figure 3) is a Java applet. Aperio viewers is a Zoomify-based plug-in with basic functions like navigation, zoom and map of the slide, and optional annotation list. Olympus web viewer was considered by all users as most user friendly, but it also has only basic functions. Aurora java applet took a mean time of 30 seconds to load the first time, and it has additional functions like navigation mode or measuring tools, additionally, it is the only viewer that can be used to add new annotations and text associated to the complete virtual slide or a specific area.

### Time results

Table 2 shows the results of the median value of the time to access overview, 10×, 20× and 40× areas of the eight virtual slides used in this study.

**Table 2:** Mean time to access 8 virtual slides by 5 pathologists

**Vendor**	**Overview**	**10×**	**20×**	**40×**
**Hospital network connection**				
Aurora	3.56	2.08	2.31	2.14
Aperio	2.92	5.96	3.16	1.98
Olympus	4.29	2.69	2.54	2.35
				
**ADSL residential network connection**				
Aurora	9.08	4.50	3.67	3.42
Aperio	4.42	7.65	6.68	3.06
Olympus	7.13	5.73	4.35	3.52

All figures in seconds.

In general, overview images took more time to show clear than high magnification images. Aperio viewer was very efficient in the presentation of the overview image, but in the Hospital (intranet) environment, the difference was only statistically significant (p = 0.05) when Aperio viewer was compared with the Olympus viewer. In residential ADSL (Internet) connections Aperio viewer was faster than Aurora viewer (p = 0.010) and it was also faster than Olympus viewer (p = 0.017) to show overview images.

Lower magnifications (10×) fields in most systems took longer to show sharp and clear then larger magnifications. Aperio web viewer was slower in showing 10× images (mean 5.96 SD 1.12 in intranet), and both in intranet and Internet; it was significantly slower than Aurora viewer (p < 0.001 and p = 0.002, respectively) and Olympus viewer (p < 0.001 and p = 0.019, respectively). The best results for 10× magnifications were obtained with Aurora viewer, which was also significantly faster (p = 0.027 and p = 0.025, respectively) than Olympus viewer.

Working with larger magnifications (20× and 40×) was considered fast by all users in all tested systems, only the Aperio system showed a trend to be slower in 20×, and faster in 40× fields, but in the intranet connection, there was no statistically significant difference between the three systems in both Intranet and Internet connections.

It is noticeable that in larger bandwidth networks, Aurora viewer shows quite homogeneous results in all magnifications.

Using Aurora viewer we did not find significant differences in the time to access Aperio SVS files and Olympus VSI files.

## Discussion

The Health Care Services of Castilla-La Mancha (SESCAM) has implemented virtual slides imaging in eight hospitals of that part of Spain [3]. A previous comparison study revealed significant differences in the technology of different whole slide imaging vendors [4].

The subjective experience of using virtual slides by pathologists can be affected by many factors on sever side and on client side. On server side we need to consider factors associated to physical resources (sever performance, disk performance and network bottlenecks) and those related to software performances like web server optimization or file accessing optimization, this is especially important in virtual slide technology. Our study took into consideration both sever and client factors, to compare similar environments. In Internet connections, our study did not control for available network bandwidth at the moment of the study nor factors associated on client side (PC performance), except for similar screen size (1024 × 768 pixels).

Efficiency and performance of studied viewers is dependant on the magnification used. Aperio viewer is very efficient in overview images. The pyramidal optimized structure (with several layers of images at different magnifications) of Aperio images [4] allows a reduction in the presentation of the overview image.

Aurora viewer is especially efficient in lower magnifications (10×). For larger magnifications (20× and 40×) no significant differences were found between different vendors.

File size (dimensions in pixels and file size in disk) did not have a significant impact in measured times.

## Conclusion

Performance information can help pathologists to decide which viewer is more suitable for their specific needs. Lower magnifications are difficult to optimize for speed in virtual slides but they are important in screening and larger biopsies studies.

## Competing interests

The authors declare that they have no competing interests.
